# Paratesticular sarcoma of the spermatic cord: A rare case of dedifferentiated leiomyosarcoma

**DOI:** 10.1016/j.eucr.2026.103372

**Published:** 2026-02-09

**Authors:** Resul Çi̇çek, İbrahim Topçu, Bulut Dural, Serhat Toprak, Ömer Faruk Yıldırım, Yasin Karaçor

**Affiliations:** a Department of Urology, Faculty of Medicine, Inonu University, Malatya, Türkiye; b Department of pathology, Faculty of Medicine, Inonu University, Malatya, Türkiye

**Keywords:** Paratesticular sarcoma, Dedifferentiated leiomyosarcoma, Radical inguinal orchiectomy, Immunohistochemistry

## Abstract

Paratesticular sarcomas are extremely rare tumors characterized by aggressive biological behavior and a significant risk of recurrence and metastasis. We present a rare case of paratesticular sarcoma ultimately diagnosed as dedifferentiated liposarcoma following comprehensive histopathological and immunohistochemical evaluation. The patient presented with complaints of scrotal edema and discomfort, and radical inguinal orchiectomy was performed. The definitive diagnosis was established through comprehensive histological and immunohistochemical evaluation and was defined as dedifferentiated liposarcoma. This report emphasizes the critical importance of comprehensive pathological evaluation and highlights total surgical excision as the primary approach in the treatment of paratesticular sarcomas due to their rarity and complex diagnosis

## 1 Introduction

Paratesticular sarcomas constitute roughly 2% of all male genitourinary cancers, predominantly originating from the spermatic cord.^1^ These tumors display a wide histological range and may clinically resemble benign disorders such lipomas, epididymal cysts, or inguinal hernias, sometimes resulting in delayed or inadvertent diagnosis.^2^^,^^3^

Leiomyosarcoma (LMS) is a rare malignant soft-tissue neoplasm derived from smooth muscle cells and is noted as the second most prevalent malignant paratesticular tumor following liposarcoma.^4^^,^^5^ It primarily impacts males in their fifties and sixties, usually manifesting as a gradually increasing, painless scrotal lump.^1^ As the tumor advances, symptoms include discomfort, compressive effects, and signs of local invasion may manifest. Dedifferentiated liposarcoma represents a high-grade subtype with aggressive behavior and a significant risk of local recurrence, particularly when arising from the spermatic cord. Accurate distinction from leiomyosarcoma is critical, as morphological overlap may lead to diagnostic misclassification without appropriate molecular or immunohistochemical confirmation^6^.

Complete surgical excision is the fundamental treatment for spermatic cord leiomyosarcoma and other malignant mesenchymal tumors. Achieving negative surgical margins may be difficult due to anatomical limitations, hence Elevating the risk of local recurrence.^5^^,^^7^

This case report details the clinical course, diagnostic methodology, and surgical outcomes of a patient diagnosed with paratesticular sarcoma reported as undifferentiated leiomyosarcoma. By synthesizing this case with previously documented data, we aim to increase clinical awareness and provide insights into the management and follow-up methods for these rare but aggressive malignancies.

## 2 Case

**Fig. 1:**
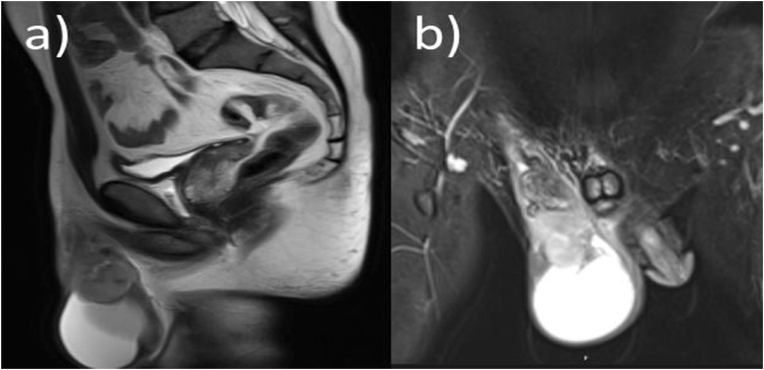
Pelvic magnetic resonance imaging demonstrating a well-defined, heterogeneous solid mass arising from the spermatic cord in the paratesticular region. The lesion shows predominantly intermediate signal intensity on T1-weighted images and heterogeneous high signal intensity on T2-weighted images, with displacement of adjacent scrotal structures and no evidence of direct testicular invasion.

A 61-year-old male patient exhibited scrotal soreness and a progressively increasing lump that had persisted for roughly 6 months. The physical examination disclosed a solid, non-painful paratesticular tumor. Imaging studies revealed a solid lesion situated in the spermatic cord, measuring approximately 7.2 × 5.0 cm (Fig. 1A–B). Thoracoabdominal computed CT revealed no indications of distant metastatic illness.

**Fig. 2:**
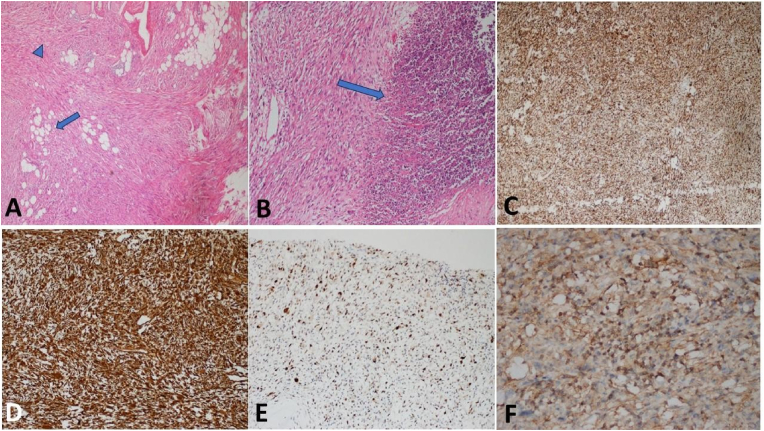
Tumoral proliferation composed of fibrosarcoma-like spindle cells arranged in intersecting fascicles (arrowhead), with scattered lipoblasts (arrow) (A, H&E × 40), areas of necrosis (arrow) (B, H&E × 100), and immunohistochemical positivity for p16 (C, × 100), vimentin (D, × 100), and nuclear MDM2 expression (E, × 100).

The patient underwent radical inguinal orchiectomy as the definitive surgical procedure. Histopathological evaluation demonstrated a densely cellular tumor composed of spindle-shaped cells arranged in intersecting fascicles, exhibiting fibrosarcoma-like architecture with scattered lipoblasts and areas of necrosis (Fig. 2A and B). Immunohistochemical analysis

showed strong positivity for p16 (Fig. 2C), vimentin (Fig. 2D), and nuclear MDM2 expression (Fig. 2E–F), confirming the diagnosis of dedifferentiated liposarcoma. Although CDK4, smooth muscle actin, and desmin are well-established diagnostic markers for liposarcoma and leiomyosarcoma respectively, these markers were not contributory in the present case and therefore were not emphasized in the diagnostic interpretation.

Despite negative surgical margins for tumor involvement, the lesion's closeness to adjacent anatomical structures constrained the attainable margin width. The postoperative period was uneventful. Due to the tumor subtype's aggressive biological behavior and significant recurrence potential, the patient was enrolled in a systematic surveillance program. No local recurrence or distant metastases were observed during a 9-month follow-up period. The patient was enrolled in a structured surveillance program consisting of regular physical examinations and cross-sectional imaging.

## 3 Discussion

Paratesticular sarcomas are uncommon malignant mesenchymal tumors originating from tissues including the spermatic cord, epididymis, and testicular tunics, representing roughly 1–2% of all male genitourinary malignancies.^4^ Owing to their gradual development and generally asymptomatic nature, these tumors are frequently misidentified as benign paratesticular diseases, such as lipomas, hernias, or epididymal cysts, potentially leading to delayed diagnosis and inadequate initial treatment.^2^

The biological behavior of paratesticular sarcomas is predominantly influenced by histological subtype and tumor grade. High-grade tumors, including dedifferentiated liposarcoma and leiomyosarcoma, have aggressive clinical trajectories, marked by elevated incidences of local recurrence and distant metastasis relative to low-grade mesenchymal tumors.^8^ Extensive institutional studies have repeatedly shown that histology, grade, tumor size, and margin status are the primary prognostic factors affecting oncological outcomes.^7^

The precise diagnosis of paratesticular sarcomas continues to be difficult despite advancements in imaging techniques. Ultrasonography and magnetic resonance imaging are effective for determining lesion location and size; however, these modalities lack adequate specificity to consistently distinguish between benign and malignant mesenchymal tumors or to anticipate histological subtype. Thus, a conclusive diagnosis depends on histological analysis post-surgical excision, highlighting the restricted utility of imaging as an independent diagnostic method.

Immunohistochemistry is essential for the categorization of paratesticular sarcomas and for differentiating them from histological mimickers. Dedifferentiated liposarcomas are distinguished by the amplification and overexpression of MDM2 and CDK4, which function as very sensitive and specific diagnostic indicators. Markers such as p16 and vimentin, while not specific, may provide supportive evidence in high-grade mesenchymal tumors and should be interpreted in conjunction with morphological features and lineage-specific markers(2). Leiomyosarcomas generally have positivity for smooth muscle markers, including smooth muscle actin and desmin^6^, ^9^.

Nonetheless, immunohistochemistry results should invariably be analyzed alongside morphological characteristics, as no singular marker is wholly unique, especially in high-grade or poorly differentiated neoplasms.

Surgical excision is the fundamental treatment for paratesticular sarcomas. Radical inguinal orchiectomy with high closure of the spermatic cord is recognized as the preferred surgical technique, facilitating en bloc resection and reducing the likelihood of tumor seeding.^1^

Numerous high-impact studies have highlighted that insufficient initial surgery, such as scrotal violation or incomplete excision, correlates with markedly elevated rates of local recurrence.^4^^,^^7^ Achieving extensive negative margins may be technically difficult due to the proximity of the tumor to critical inguinal structures.

The function of adjuvant therapy in paratesticular sarcomas is still contentious. Retrospective results indicate that adjuvant radiation may diminish local recurrence in high-grade tumors or instances with narrow or positive surgical margins, however a definitive survival advantage has not been consistently established^7^ Chemotherapy is often designated for metastatic or unresectable conditions, with limited and inconsistent efficacy in genitourinary soft tissue sarcomas.^3^

Due to the significant risk of local recurrence and delayed metastasis—especially in dedifferentiated liposarcoma and high-grade leiomyosarcoma—prolonged follow-up is necessary. Previous series have reported that local recurrence of dedifferentiated liposarcoma most commonly occurs within 24–36 months following surgery, although late recurrences beyond five years have also been described. Prior research has indicated local recurrence rates of 40–50% for dedifferentiated liposarcomas, with the lungs being the predominant location of distant metastasis.^6^^,^^10^ 5Consequently, prolonged monitoring with regular cross-sectional imaging is highly advised, even for patients with initially positive surgical results.

In conclusion, paratesticular sarcomas are uncommon yet potentially malignant neoplasms

that necessitate a heightened level of suspicion for prompt identification. Complete surgical excision is the principal therapeutic approach, whereas histological and immunohistochemical evaluations are essential for precise tumor categorization. Due of the potential for late recurrence and metastasis, systematic long-term follow-up is essential to enhance oncological outcomes. Thoroughly documented case reports and short series are crucial in enhancing diagnostic and therapeutic approaches for these rare cancers.

## CRediT authorship contribution statement

**Resul Çi̇çek:** Writing – review & editing. **İbrahim Topçu:** Writing – original draft. **Bulut Dural:** Data curation. **Serhat Toprak:** Data curation. **Ömer Faruk Yıldırım:** Data curation. **Yasin Karaçor:** Data curation.
